# Overexpression of *HbMBF1a*, encoding multiprotein bridging factor 1 from the halophyte *Hordeum brevisubulatum*, confers salinity tolerance and ABA insensitivity to transgenic *Arabidopsis thaliana*

**DOI:** 10.1007/s11103-019-00926-7

**Published:** 2019-10-26

**Authors:** Lili Zhang, Yunxiao Wang, Qike Zhang, Ying Jiang, Haiwen Zhang, Ruifen Li

**Affiliations:** 1 Beijing Academy of Agriculture and Forestry Sciences, No. 9 Shuguang Huayuan Middle Road, Haidian District, Beijing, 100097 China; 2 Beijing Key Laboratory of Agricultural Genetic Resources and Biotechnology, Beijing Agro-Biotechnology Research Center, Beijing, 100097 China; 3 College of Life Science, Hebei Normal University, Shijiazhuang, 050024 China

**Keywords:** *HbMBF1a*, STEM (short time-series expression miner), *Hordeum brevisubulatum*, Transgenic *Arabidopsis thaliana*, Salt and ABA treatments

## Abstract

**Key message:**

*HbMBF1a* was isolated and characterized in *H. brevisubulatum*, and overexpressed *HbMBF1a* could enhance the salt tolerance and ABA insensitivity in *Arabidopsis thaliana*. The transcript levels of stress-responsive genes were significantly increased in the transgenic lines under salt and ABA conditions.

**Abstract:**

Salinity is an abiotic stress that considerably affects plant growth, yield, and distribution. *Hordeum brevisubulatum* is a halophyte that evolved to become highly tolerant to salinity. Multiprotein bridging factor 1 (MBF1) is a transcriptional coactivator and an important regulator of stress tolerance. In this study, we isolated and characterized *HbMBF1a* based on the transcriptome data of *H. brevisubulatum* grown under saline conditions. We overexpressed *HbMBF1a* in *Arabidopsis thaliana* and compared the phenotypes of the transgenic lines and the wild-type in response to stresses. The results indicated that *HbMBF1a* expression was induced by salt and ABA treatments during the middle and late stages. The overexpression of *HbMBF1a* in *A. thaliana* resulted in enhanced salt tolerance and ABA insensitivity. More specifically, the enhanced salt tolerance manifested as the increased seed germination and seedling growth and development. Similarly, under ABA treatments, the cotyledon greening rate and seedling root length were higher in the *HbMBF1a*-overexpressing lines, suggesting the transgenic plants were better adapted to high exogenous ABA levels. Furthermore, the transcript levels of stress-responsive genes were significantly increased in the transgenic lines under salt and ABA conditions. Thus, *HbMBF1a* is a positive regulator of salt and ABA responses, and the corresponding gene may be useful for producing transgenic plants that are salt tolerant and/or ABA insensitive, with few adverse effects. This study involved a comprehensive analysis of *HbMBF1a*. The results may provide the basis and insight for the application of *MBF1* family genes for developing stress-tolerant crops.

**Electronic supplementary material:**

The online version of this article (10.1007/s11103-019-00926-7) contains supplementary material, which is available to authorized users.

## Introduction

Salt stress is a serious threat affecting crop production and distribution. High salinity, especially during the seedling stage, dramatically decreases crop yield and quality, and may result in the death of all seedlings. Therefore, the main objective of this study was to functionally characterize salt-responsive genes in the halophyte *Hordeum brevisubulatum* and use them for breeding halotolerant crops. Previous studies have identified and described the salt-tolerance genes cloned from model plant species such as *Arabidopsis thaliana* (Kim et al. 2007; Alavilli et al. 2017).

*Hordeum brevisubulatum*, which is a wild relative of cultivated barley in the genus *Hordeum* in the tribe *Triticeae*, is a typical clonal plant of the grass family with a short rhizome. The salient feature of *H. brevisubulatum* is its high tolerance to multiple abiotic stresses, including salinity, alkalinity and drought, enabling it to grow in the saline-alkali grasslands of northern China, where it is used as a major forage crop for livestock (Li et al. 2012). Although previous studies have investigated *H. brevisubulatum* regarding ion balance and gene functions (Zhang et al. 2015), very little is known about the molecular mechanism underlying the resistance of *H. brevisubulatum* to stress.

Transcriptional coactivators are important for the expression of eukaryotic genes because they represent the link between transcription factors/other regulatory elements and the basic transcriptional machinery (Takemaru et al. 1997). For example, multiprotein bridging factor 1 (MBF1) is a highly conserved transcriptional coactivator that influences diverse processes. The MBF1 proteins of various organisms are linked to the TATA-binding protein via interactions with C-Jun, GCN4, ATF1, or other nuclear receptors to activate gene transcription (Brendel et al. 2002). Plant MBF1s are involved in growth and development as well as a variety of stress responses. The overexpression of *MBF1* may regulate diverse signal transduction pathways and activate the production of multiple defense factors, ultimately increase plant resistance to multiple stresses (Arce et al. 2010; Wang et al. 2017).

The *MBF1* gene is likely one of the primary targets of physiological signals in plants. The functions and types of MBF1 have been reported for *A. thaliana* as a model plant (Tsuda and Yamazaki 2004). A previous study confirmed that MBF1 can control the leaf cell cycle and leaf expansion in *A. thaliana*, and that has been divided three subtypes, namely AtMBF1a, AtMBF1b and AtMBF1c. AtMBF1c is a positive regulator of seed germination and plant growth (Hommel et al. 2008). Other investigations indicated there are no growth rate differences between *AtMBF1a*-overexpressing and wild-type (WT) lines (Kim et al. 2007), whereas the overexpression of *AtMBF1c* results in a 20% increase in seed yield (Suzuki et al. 2005). These studies suggest that *MBF1c* likely evolved differently than *MBF1a* and *MBF1b*, and that *MBF1c* has a non-redundant function. In addition, The *A. thaliana mbf1* triple knock-down mutant (*mbf1 abc*-) is hypersensitive to oxidative and osmotic stresses, and the phenotypes are either partially or fully restored by *AtMBF1c* overexpression, implying AtMBF1c is the predominant MBF1 subtype responsible for stress tolerance (Arce et al. 2010).

Though the preliminary progress was made in the *MBF1* genes associated with plant tolerance to abiotic and biotic stresses, most plant species produce only one type of MBF1 protein (Tsuda and Yamazaki 2004). Moreover, these *MBF1* genes were all cloned from the common plants, but not specific plants tolerant to stresses. The expression of wheat *MBF1* may be induced by salicylic acid and ethephon stresses (Zhang et al. 2009). The phosphorylation of StMBF1 is induced in potato cells in response to a fungal treatment (Zanetti et al. 2003). In tobacco, the simultaneous exposure to high temperature and drought stresses can up-regulate *MBF1* expression (Rizhsky et al. 2002). Tomato mosaic virus mobile proteins and proteins from related viruses can interact with MBF1 to regulate host gene expression (Matsushita et al. 2002).

To investigate the functional advantages of *H. brevisubulatum* genetic resources, we performed the transcriptome sequencing and found that it had three subtypes and these three subtypes were differentially produced in development and various tissues. So the salt stress-responsive *HbMBF1a* gene was isolated this halophytic grass based on transcriptome data. Moreover, we functionally characterized HbMBF1a under salt and abscisic acid (ABA) treatments by gene overexpression and stress-related gene expression analyses. The *HbMBF1a* gene may be useful for the cultivation of new highly salt-tolerant transgenic crops, but the underlying mechanism and function of HbMBF1a remain relatively unclear. Therefore, investigating the *MBF1* gene family is important for revealing its specific role and the related regulatory mechanism associated with plant stress resistance.

## Materials and methods

### *Hordeum brevisubulatum* material and stress treatments

*Hordeum brevisubulatum* plants were collected from the saline grassland region of Hohhot, which is in the Inner Mongolia Autonomous Region of China. The collected plants were screened for salt tolerance. The seeds were vernalized by immersing in water for 2 days at 4 °C, after which they were incubated at 22–25 °C under a 16-h light/8-h dark photoperiod for 2–3 days to promote germination. When the resulting seedlings grew to about 1.0 cm long, they were transferred to a 250-ml beaker containing Hoagland’s nutrient solution. The seedlings were then cultured for approximately 2 weeks until the plants reached the two-leaf and one-heart stage, at which point they were treated with 350 mM NaCl or 20 μM ABA.

For an RNA-sequencing analysis, shoot and root samples were collected at 0, 1, 6, and 24 h after the NaCl treatment, and immediately frozen in liquid nitrogen and stored at − 80 °C. To examine gene expression profiles, plants were exposed to NaCl or ABA treatment for 0, 0.5, 1, 2, 3, 6, and 12 h, after which shoot and root samples were collected and immediately frozen in liquid nitrogen and stored at − 80 °C.

### Genetic transformation and stress treatments of *Arabidopsis thaliana*

The pYBA1104-35S:*HbMBF1a* construct in *Agrobacterium tumefaciens* GV3101 was transformed into *A. thaliana* ecotype Colombia-0 (WT) plants via the floral dip method. The infiltration medium comprised 2.2 g/l Murashige & Skoog Basal Medium with Vitamins (PhytoTechnology Laboratories, USA), 50 g/l sucrose, and 0.2 ml/l Silwet-77, with a pH of 5.7–5.8 (Clough and Bent 1998). The *HbMBF1a*-overexpressing transgenic lines (T_1_ generation) were selected with kanamycin (Solarbio, China), and lines with one *HbMBF1a* copy were identified based on a 3:1 segregation ratio for the kanamycin-resistance marker. We obtained T_3_ generation homozygous lines when all of the seedlings generated from the seeds of the transgenic lines were green during the screening for kanamycin resistance. Finally, three transgenic lines (L17, L18, and L23) were identified based on an analysis of *HbMBF1a* expression and the phenotype of the transgenic plants. These lines were analyzed as subsequently described.

For phenotypic investigations, seeds of the transgenic lines (L17, L18, and L23) and the WT control were sown on MS medium and MS medium containing 125 mM NaCl, 150 mM NaCl, 0.5 μM ABA, 1 μM ABA, or 1.5 μM ABA. Nine days later, the germination and cotyledon greening rates were determined. To analyze growth and development, the seeds of the transgenic lines (L17, L18, and L23) and the WT control were sown on MS medium for a 4-day vertical culture. Consistently growing seedlings were transferred to MS medium supplemented with 150 mM NaCl, 175 mM NaCl, 0.5 μM ABA, or 1 μM ABA, and then grown as a vertical culture for 8 days. The fresh weight and root length of the plants were then measured. To evaluate the salt tolerance during the adult stage, the three transgenic lines (L17, L18, and L23) and the WT control were seeded in a 35.5 cm × 28 cm × 7.6 cm culture tray containing peat soil and vermiculite (1:1). When the seedlings reached the six-leaf stage, they were treated twice with 300 mM NaCl for a total of 23 days. After resuming normal watering for an additional 7 days, the plant survival rate was calculated and plants were photographed.

To investigate stress-related gene expression, the *HbMBF1a*-overexpressing transgenic lines (L17, L18 and L23) and the WT control were grown vertically on MS medium for 7 days, after which the consistently growing seedlings were transferred to MS medium supplemented with 150 mM NaCl or 20 μM ABA. Samples were collected from the seedlings after 1 days.

### RNA isolation and cDNA synthesis

The collected plant samples were ground to a fine powder in liquid nitrogen. Total RNA was extracted from the ground material with TRIzol reagent (Takara, Dalian, China). Specifically, the powdered samples were resuspended in TRIzol reagent and then mixed by vortexing. Samples were centrifuged at 12,000×*g* for 5 min at 4 °C. Phenol–chloroform equal to one-fifth of the total volume was added to the supernatant and the resulting solution was centrifuged at 12,000×*g* for 5 min at 4 °C. An equal volume of chloroform was added and the solution was centrifuged at 12,000×*g* for 5 min at 4 °C, after which a half volume of 8 M LiCl and a half volume of 75% alcohol were added to the supernatant to precipitate the RNA for at least 1 h. Finally, 75% ethanol was used to purify the RNA, which was finally dissolved in RNAase-free water (Zhang et al. 2018). The RNA concentration was determined with the NanoDrop 2000c UV–Vis spectrophotometer (Thermo Fisher Scientific Inc., Waltham, MA, USA).

The total RNA was used as the template for the synthesis of cDNA with the HiScript II Q Select RT SuperMix reagent Kit (Vazyme Biotech Co., Ltd, Nanjing, China). The cDNA templates were diluted 1:5 with nuclease-free water and stored at − 20 °C until analyzed in a quantitative real-time polymerase chain reaction (qRT-PCR) assay with minimal thawing and refreezing.

### Illumina library preparation and sequencing data analysis

The preparation of transcriptome libraries and deep sequencing were performed by Beijing Ori-Gene Science and Technology CoRP., LTD (Beijing, PR China). Transcriptome libraries were constructed using Ribo-Zero-rRNA Removal Kits (Plant/Bacteria) (Illumina, San Diego, CA, USA) and NEBNext® Ultra™ RNA Library Prep Kit for Illumina (New England Biolabs)according to the manufacturer’s instructions. Libraries were controlled for quality and quantified using the BioAnalyzer 2100 system (Agilent Technologies, CA, USA) and qPCR (Kapa Biosystems, Woburn, MA, USA). The resulting libraries were sequenced initially on HiSeq2500 instrument that generated paired-end reads of 125 nucleotides.

Raw data of fastq format were firstly processed as follows. Adapter sequences, primers, Ns, and reads with quality scores below 30 were trimmed. Reads with a length < 60 bp after trimming were discarded. In this step, clean data were obtained. The read1 files from all samples were pooled into one big read1.fq file, and read2 files into one big read2.fq file. Transcriptome assembly was accomplished based on the read1.fq and read2.fq using Trinity (Grabherr et al. 2011).

### Differential genes expression analysis and time-series expression profile clustering

In analysis, a criterion of |log2 (fold-change)| ≥ 1 and P value ≤ 0.05 between the two conditions was used to identify differentially expressed genes (DEG). DEG were set as the foreground and all of the transcripts as the background, Hyper-geometric distribution was employed to detect the significant GO terms and KEGG pathways at a significance level of 0.05.

Hierarchical cluster analysis was performed using transcriptomic data from groups. Heatmap.2, an R package, was used to show genes’ heatmap. The heatmap represents cluster analysis of differentially expressed unigenes/genes according to gene expression level.

We used STEM software (Short Time-series Expression Miner, version 1.3.11) to analyze abundance changes of differentially expressed unigenes (Ernst and Bar-Joseph 2006). STEM implements a novel clustering method that can identify significant temporal expression profiles. The gene clusters were ranked by the p value significance of the observed number of genes that fit a profile beyond the expected number (Jing et al. 2011).

### Quantitative real-time PCR

A qRT-PCR assay was completed with TB Green™ Premix Ex Taq™ II (Takara, Dalian, China) and the StepOne Plus Real-Time PCR System (Applied Biosystems, Foster City, CA, USA). The 20-µl reactions were performed in MicroAmp Fast Optical 96-well reaction plates with barcodes (Applied Biosystems), with each well containing 2 μl cDNA, 10 μl 2 × qPCR Mix, 0.4 μl 50 × ROX reference dye, 0.8 μl forward and reverse primers, and 6 μl nuclease-free water. The PCR program was as follows: 95 °C for 30 s; 40 cycles of 95 °C for 5 s and 60 °C for 30 s, during which the fluorescent signal was detected.

The expression levels of the following 13 stress response-related genes were analyzed: *AtRD29A* (*Responsive to desiccation 29A*), *AtRD29B* (*Responsive to desiccation 29B*), *AtRD22* (*Responsive to dehydration 22*), *AtRAB18* (*RAB GTPase homolog B18*), *AtCOR47* (*Clod*-*regulated 47*), *AtERD11* (*Early response to dehydration 11*), *AtDREB2A* (*DRE*-*Binding protein 2A*), *AtABI3* (*ABA insensitive 3*), *AtABI4* (*ABA insensitive 4*), *AtABI5* (*ABA insensitive 5*), *AtNCED3* (*9*-*cis*-*epoxycarotenoid dioxygenase 3*), *AtKIN1* (*Kinase 1*), and *AtWIN1* (*Wax inducer 1*). Details regarding the primers designed for these target genes are provided in Supplementary Table S1. We selected *H. brevisubulatum* reference genes and designed the corresponding primers as previously described (Zhang et al. 2018).

### Sequence analysis, phylogenetic analysis, subcellular localization, and transcriptional activation of HbMBF1a

Amino acid sequences were analyzed for the presence of the expected MBF1 domains with the NCBI (https://www.ncbi.nlm.nih.gov/) and SMART (http://smart.embl-heidelberg.de/) online tools. Multiple sequences were aligned with the ClustalW (http://www.ch.embnet.org/software/ClustalW.html) and BioEdit (http://www.mbio.ncsu.edu/BioEdit/bioedit.html) programs. We identified MBF1 proteins from various organisms via alignments with the sequences in the NCBI database. These MBF1 proteins as well as the three *H. brevisubulatum* MBF1 proteins were used to construct a phylogenetic tree with the MEGA 6.0 software (Higgins et al. 1996). The three AtMBF1 proteins were derived from the TAIR database (https://www.arabidopsis.org/), whereas the MBF1a proteins of gramineous crops were retrieved from the EnsemblPlants database (http://plants.ensembl.org/hmmer/index.html).

A cDNA fragment comprising the *HbMBF1a* open reading frame (stop codon removed) was amplified by PCR with a pair of primers containing *Bam*HI or *Sac*I sites (Supplementary Table S1). The PCR products were digested with *Bam*HI or *Sac*I and then cloned into the pGreen0029-GFP vector so the *HbMBF1a* coding sequence was fused in frame with the sequence encoding the green fluorescent protein (GFP) for expression under the control of the cauliflower mosaic virus 35S promoter. Because *AtCBF1* is a nuclear localization gene, a positive control vector was constructed for the production of the AtCBF1-red fluorescent protein (RFP) fusion protein, which was used to determine the location of the nucleus. The recombinant pGreen-35S-*HbMBF1a*-*GFP* vector and the positive control vector were inserted into *A. thaliana* mesophyll protoplasts as previously described (Yoo et al. 2007). The fluorescent signals were detected by fluorescence microscopy.

The *HbMBF1a* open reading frame was cloned into the pGBKT7 vector (Clontech Laboratories, Inc., USA) using gene-specific primers (Supplementary Table S1). The empty pGADT7 plasmid and pGBKT7-*HbMBF1a* recombinant plasmid were used to co-transform yeast AH109 cells to verify the transcriptional activation by HbMBF1a. The co-transformation with pGBKT7-53 and pGADT7-T served as a positive control, whereas the co-transformation with pGBKT7-Lam and pGADT7-T was used as a negative control. The transcriptional activation by HbMBF1a was evaluated based on the growth characteristics of the transformants on SD medium (-Leu/-Trp and -Leu/-Trp/-His). The β-galactosidase activity was detected with X-β-Gal. The yeast two-hybrid assay was performed with the Matchmaker™ Gold Yeast Two-Hybrid System (Clontech Laboratories, Inc.).

### Statistical analysis

All experiments were repeated at least three times and the data are presented herein as the mean ± standard deviation. Microsoft® Excel 2016 and the SAS 9.2 software were used for analyzing data and conducting Student’s *t*-test, respectively. The thresholds for the significance of the differences among samples were as follows: P < 0.05 with *, P < 0.01 with **, and P < 0.001 with ***.

## Results

### Transcriptome data analysis of *H. brevisubulatum* exposed to salt stress

To elucidate the salt-tolerance mechanism of the halophyte *H. brevisubulatum*, we exposed plants to salt stress and then sequenced the transcriptome of the shoots and roots at various time-points (0, 1, 6, and 24 h after the salt stress treatment). For each condition, three independent biological replicates were analyzed. The RNA-sequencing libraries were sequenced with the Illumina HiSeq 2500 System, with 125 bp reads. After removing low quality sequences, adapter and barcode sequences, and possible viral and rRNA contaminated reads, a total of 72,066,413 clean reads were obtained. The de novo assembly of these high-quality clean reads generated 82,431 unique transcripts and 59,032 unigenes, with an average length of 810 bp and 732 bp, respectively. The longest transcript was 15,516 bp and the N50 was 1293 bp.

**Fig. 1:**
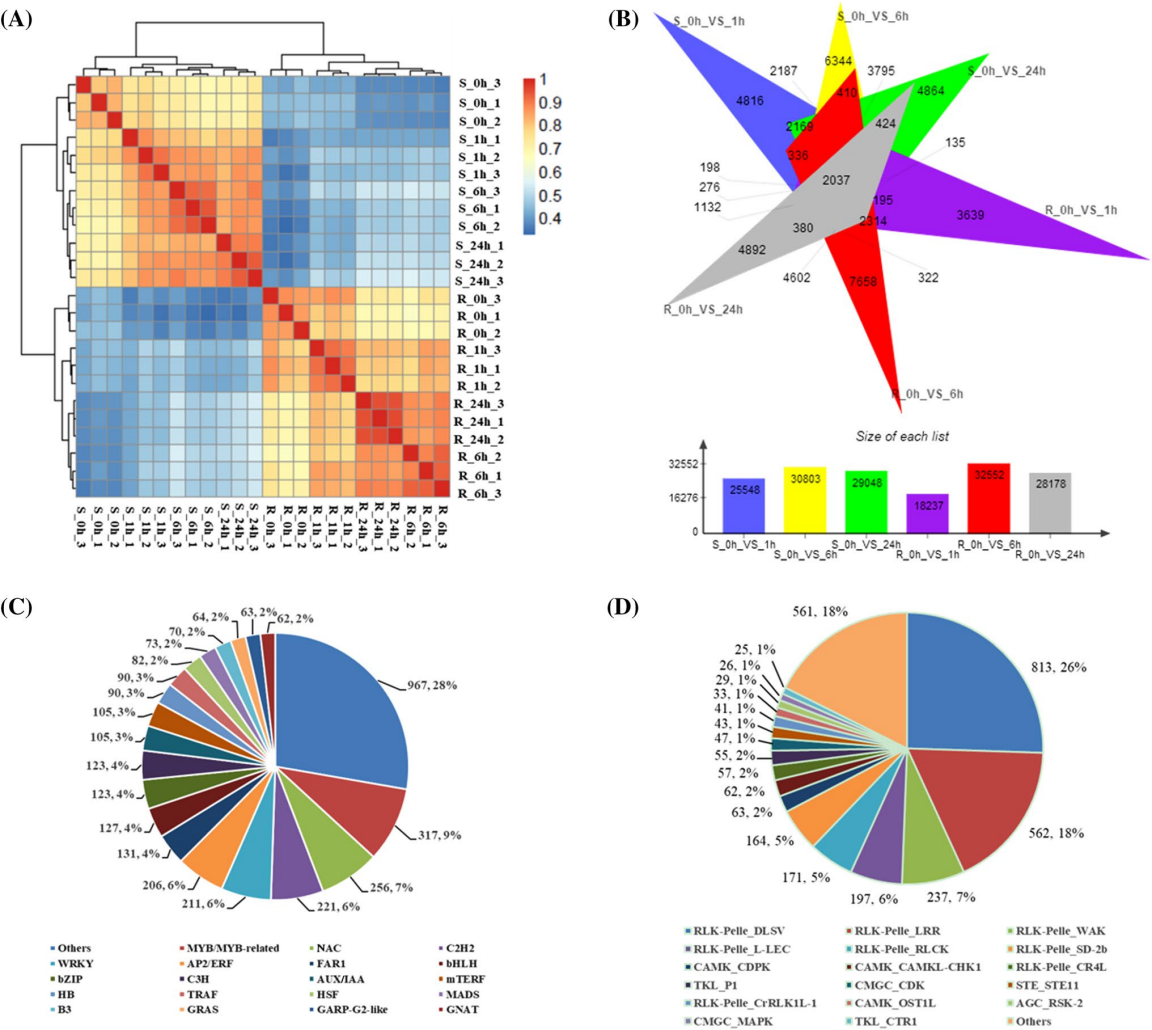
Transcriptome data analysis of *H. brevisubulatum* plants exposed to salt stress. **a** Heat map revealing the correlation between samples. The horizontal and vertical coordinates provide the sample names, and the color represents Pearson’s correlation coefficient (*r*). The closer *r* is to 1, the stronger the correlation between the two samples. **b** Comparison of the differentially expressed genes among samples due to salt stress. Blue, yellow, and green indicate the differentially expressed genes in the *H. brevisubulatum* shoots after a salt treatment for 1, 6, and 24 h, respectively, relative to the expression levels at 0 h. Purple, red, and gray represent the differentially expressed genes in the *H. brevisubulatum* roots at 1, 6, and 24 h, respectively, relative to the expression levels at 0 h. **c** Number and classification of unique transcripts annotated as a transcription factor in the transcriptome of salt-treated *H. brevisubulatum*. **d** Number and classification of protein kinases identified in the transcriptome of salt-treated *H. brevisubulatum*

Pearson’s correlation coefficient (*r*) is an indicator of the correlation between biological replicates. The closer *r* is to 1, the stronger the correlation between two repeated samples. The *r* of each sample was calculated in pairs, and a gene expression heat map was drawn (Fig. 1a). The various tissues and stress treatment time-points of samples were clustered separately, and three biological replicates were clustered together, indicating that the samples used for sequencing were consistent and that there was no problem with the treatments. Venn diagrams for the six combinations of the comparison of the differentially expressed genes revealed the number of shared and unique differentially expressed genes in the *H. brevisubulatum* shoots and roots in response to salt stress for specific treatment periods (Fig. 1b).

Transcription factors and protein kinases are important upstream regulatory proteins that are critical for various plant developmental processes and responses to abiotic and biotic stresses. In the present study, we identified 3485 transcription factors that were classified into 72 families as well as 3186 protein kinases belonging to 62 families (Fig. 1c, d). The largest group of transcription factors was from the MYB family (967, 27.7%), followed by the NAC (256, 7.3%), C2H2 (221, 6.3%), WRKY (211, 6.1%), and AP2/ERF (206, 5.9%) families. These five families represented approximately half of the transcription factors identified among the unique *H. brevisubulatum* transcripts (Fig. 1c). The most abundant group of protein kinases belonged to the receptor-like protein kinase family (2623, 82.3%), which included members of the DLSV subfamily (813, 25.5%), leucine-rich repeat receptor kinases (LRR subfamily) (562, 17.6%), wall-associated kinase-like kinases (WAK subfamily) (237, 7.4%), legume lectin domain kinases (L-LEC subfamily) (197, 6.2%), receptor-like cytoplasmic kinases (RLCK subfamily) (171, 5.4%), and other receptor-like protein kinases (643, 20.2%). Other abundant groups included S-domain kinases (SD-2b subfamily) (164, 5.1%). The transcriptome dataset also included a few other protein kinases, such as calcium-dependent protein kinases (CDPK) (63, 2%), checkpoint kinase 1 (CHK1) (62, 1.9%), and MAPKs (26, 1%), which are reportedly important for plant responses to abiotic stresses (Ludwig et al. 2004; Rodriguez et al. 2010) (Fig. 1d). These data would display the transcriptome changes and provide a clue for *H. brevisubulatum* in response to salt stress.

### Screening of the differentially expressed genes in the transcriptome to identify *HbMBF1a*

**Fig. 2:**
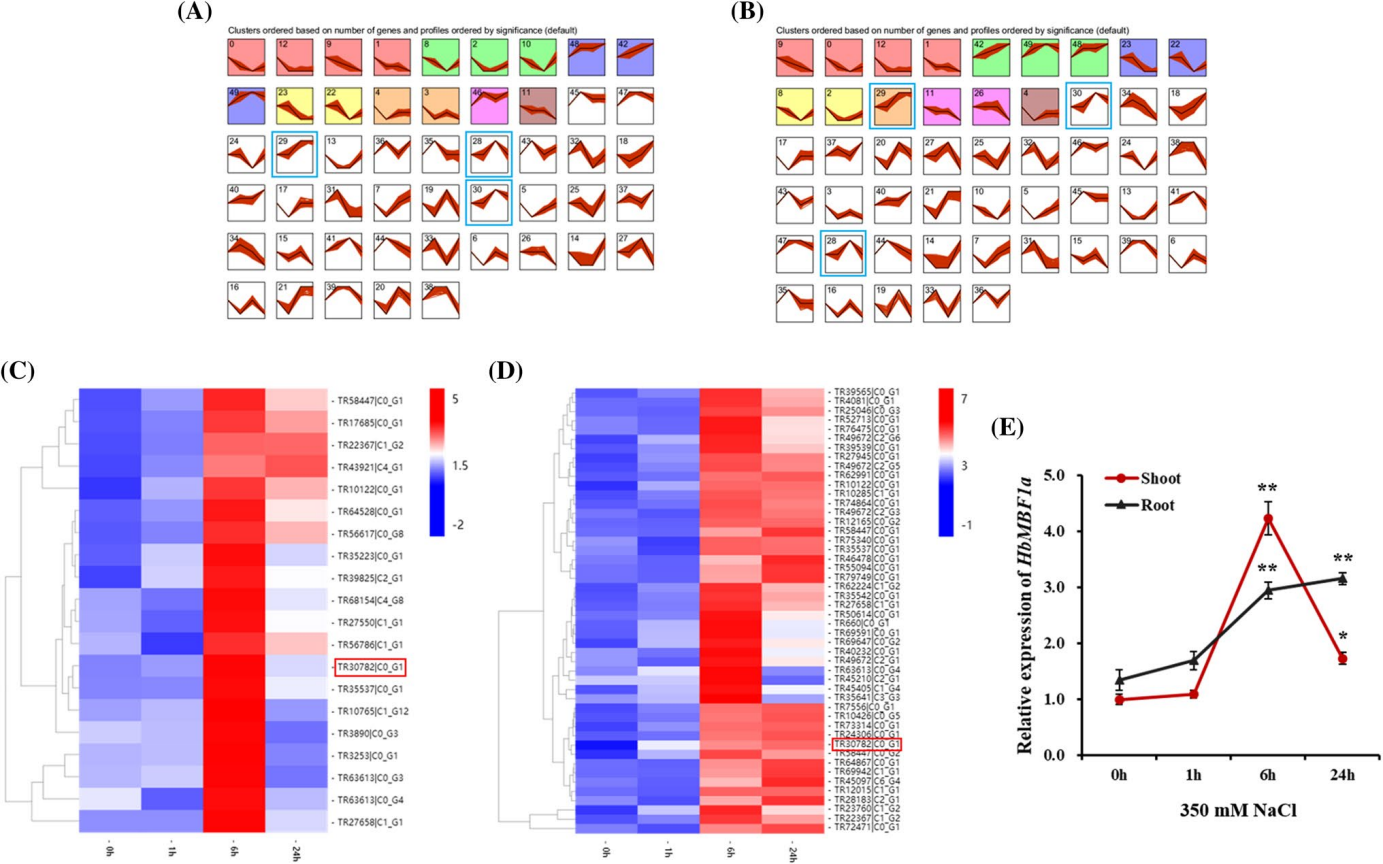
Analyses of the expression patterns of differentially expressed genes identified in the transcriptome of *H. brevisubulatum* under salt stress conditions. The main model expression profiles of differentially expressed genes in the transcriptomes of *H. brevisubulatum* shoots (**a**) and roots (**b**) after a salt treatment. Each box corresponds to a model expression profile. Colored profiles include a significant number of genes. Heat map of the expression of salt stress-responsive transcription factor genes in profiles 28, 29, and 30 of the shoots (**c**) and roots (**d**). The *HbMBF1a* gene is indicated with a red box. **e** Relative expression of *HbMBF1a* in the transcriptome of *H. brevisubulatum* under salt stress conditions. The bar indicates the standard deviation (SD). The data are presented as the mean ± SD. *Significant differences between 0 h and the treatment time-points (P < 0.05); **extremely significant differences between 0 h and the treatment time-points (P < 0.01)

To identify the key salt stress-responsive genes in the *H. brevisubulatum* transcriptome, we used the short time-series expression miner (STEM; version 1.3.11) program to analyze the shoot (Fig. 2a) and root (Fig. 2b) expression profiles of all differentially expressed genes at various time-points. Compared with the expression levels at 0 h, a total of 47,605 genes were differentially expressed in the shoots at 1, 6, and 24 h under salt stress conditions. The expression profiles of these genes were collected in 50 model expression profiles, 16 of which included a significant number of genes (Fig. 2a). Similarly, the union of 46,635 differentially expressed genes were identified in the roots at 1, 6, and 24 h under salt stress conditions. The expression profiles of these genes were also collected in 50 model expression profiles, 15 of which comprised a significant number of genes (Fig. 2b).

Among the 50 model expression profiles, we were especially interested in profiles 28, 29, and 30, which included genes with up-regulated expression levels in the middle and late stages of the salt stress treatment. In these three profiles, we screened for transcription factors that were encoded by unigenes longer than 400 bp and that had been annotated and described. Heat maps were prepared to analyze the expression of these genes (Fig. 2c, d). Profiles 28, 29, and 30 consisted of 870 genes that were differentially expressed in the *H. brevisubulatum* shoot tissue. We analyzed the heat map of 20 transcription factor genes that met the above conditions (Fig. 2c, Supplementary Table S2). Regarding the *H. brevisubulatum* root tissue, profiles 28, 29 and 30 included 2517 genes that were differentially expressed. Additionally, a heat map of 48 transcription factor genes that satisfied the above conditions was prepared (Fig. 2d, Supplementary Table S3). A comparison of the transcription factor genes expressed in the shoot and root tissues revealed seven common genes. The annotation details for these seven genes indicated that three of the genes were responsive to both salt and ABA. Finally, we decided to perform functional analysis and validation of the *HbMBF1a* gene in these three genes, and the *HbMBF1a* unigene ID is indicated with a red box in Fig. 2c, d. The *HbMBF1a* relative expression level was significantly up-regulated in the middle (6 h) and late (24 h) stages of the salt stress treatment (Fig. 2e).

### Sequence comparison and phylogenetic analysis of MBF1 proteins

**Fig. 3:**
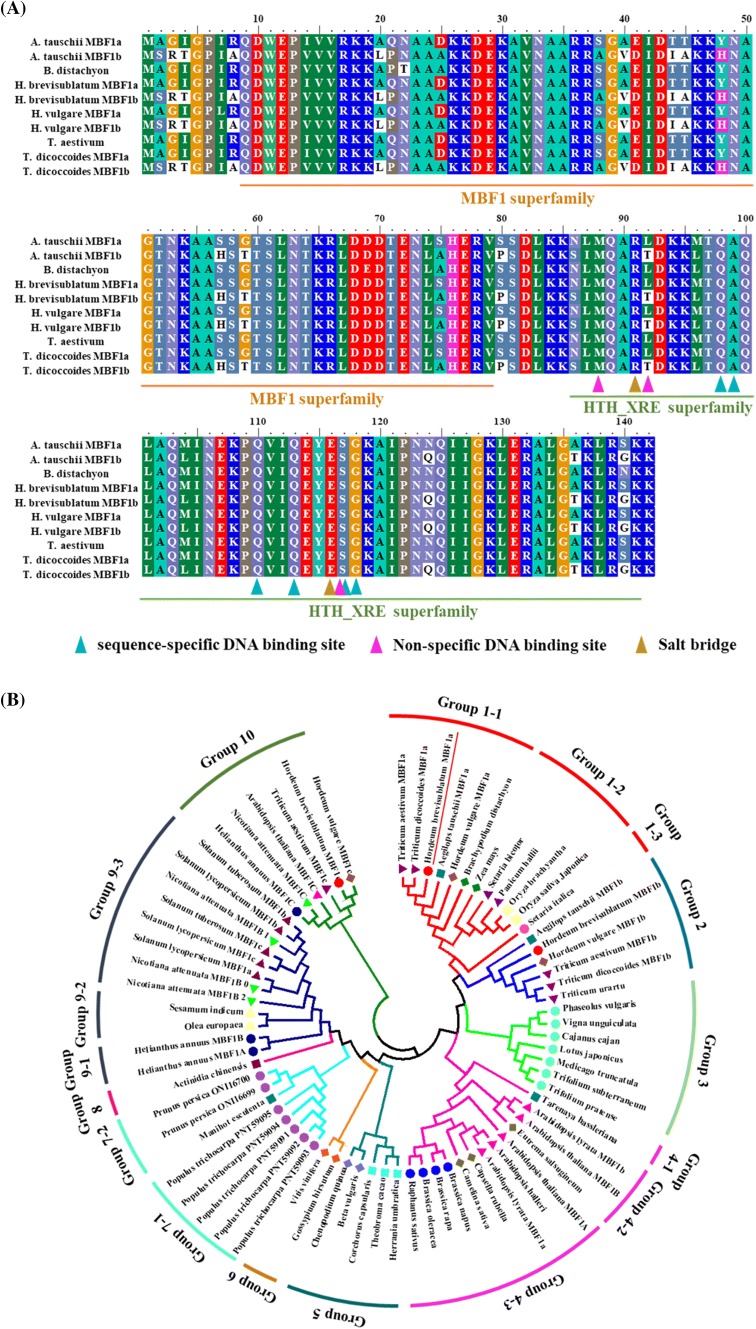
Comparison of the deduced HbMBF1a amino acid sequence with MBF1 protein sequences from other plant species. **a** Multiple sequence alignment of HbMBF1a conserved domains with MBF1 proteins from other plant species. The sequence-specific DNA-binding site, non-specific DNA-binding site, and salt bridge are indicated with a colored triangle. Superfamily domains are presented with colored bars underneath the aligned sequences. **b** Phylogenetic analysis of HbMBF1 s and 69 MBF1 proteins obtained from the NCBI database. Phylogenetic relationships among MBF1 protein sequences were determined with the neighbor-joining method of MEGA 6.0. The HbMBF1 proteins are indicated with red dots, and HbMBF1a is also marked with a red line

Multiprotein bridging factor 1 is a transcriptional coactivator with a C-terminal xenobiotic response element (XRE) family DNA-binding helix-turn-helix (HTH) domain. To investigate the function of *HbMBF1a* during the response of *H. brevisubulatum* to salt stress, we sequenced the *HbMBF1a* gene identified in the transcriptome (Supplementary Table S4). Unigenes TR30782|c0_g1, TR18778|c0_g1, and TR52713|c0_g1 encoded three putative MBF1 family proteins, namely *HbMBF1a*, *HbMBF1b*, and *HbMBF1c*, respectively. These unigenes were identified in an in-house *H. brevisubulatum* transcriptome database. Additionally, the *HbMBF1a* expression level tended to increase at 6 and 24 h after initiating the salt stress treatment (Fig. 2e). In contrast, the shoot and root *HbMBF1b* expression levels were not induced (Supplementary Fig. S1). However, in response to salt stress, the expression of *HbMBF1c* was down-regulated in the shoots, whereas it exhibited an upward trend in the roots (Supplementary Fig. S2). Therefore, we focused on the functional analysis of *HbMBF1a*. Unigene TR30782|c0_g1 was predicted to encode a protein with a conserved MBF1 superfamily domain comprising 71 amino acids (from position 9 to 79). Another conserved domain was identified as the HTH_XRE superfamily domain, which consisted of 56 amino acids (from position 86 to 141) and included sequence-specific DNA-binding sites, a non-specific DNA-binding site, and salt bridges (Fig. 3a).

Phylogenetic trees provide valuable information regarding the evolution of various genes or proteins from a common ancestor. They remain powerful tools for investigations of structural classifications and biological diversity as well as for providing insights into the events occurring during genetic evolution (Gregory 2008). In this study, 69 MBF1 proteins were identified in the NCBI database, and three putative MBF1 family proteins (HbMBF1a, HbMBF1b, and HbMBF1c) were detected in the *H. brevisubulatum* transcriptome. These 72 MBF1 proteins were aligned according to the neighbor-joining method in ClustalW (Fig. 3b). The resulting phylogenetic tree revealed that the 72 MBF1 proteins were clustered into 10 groups. Specifically, the MBF1a proteins from *Triticum aestivum*, *Triticum dicoccoides*, *Hordeum brevisubulatum*, *Aegilops tauschii*, *Hordeum vulgare*, and *Brachypodium distachyon* were clustered in group 1-1, whereas the corresponding proteins in *Oryza brachyantha*, *Oryza sativa*, *Panicum hallii*, *Setaria bicolor*, and *Zea mays* were clustered in group 1-2. Additionally, group 1-3 comprised only the *Setaria italica* MBF1 protein. Moreover, the MBF1b proteins from *Triticum dicoccoides*, *Triticum urartu*, *Triticum aestivum*, *Hordeum vulgare*, *Hordeum brevisubulatum*, and *Aegilops tauschii* were clustered in group 2. These results suggested that the MBF1 proteins of monocots are clustered together and are mainly concentrated in groups 1 and 2. Exceptions include *Triticum aestivum* MBF1c, *Hordeum vulgare* MBF1c, and *Hordeum brevisubulatum* MBF1c, which were clustered in group 10. The MBF1 proteins of legumes were mainly clustered in group 3, unlike the MBF1 proteins of cruciferous plants, which were mainly aggregated in group 4. Group 5 consisted of the MBF1 proteins from chenopodiaceous plants (*Chenopodium quinoa* and *Beta vulgaris*) and Malvales plants. The xylophyta MBF1 proteins were mainly concentrated in group 7, whereas the *Vitis vinifera* and *Gossypium hirsutum* MBF1 proteins were clustered in group 6. The *Actinidia chinensis* MBF1 protein was the only member of group 8. The MBF1 proteins of Solanaceae plants were mainly clustered in group 9. Furthermore, the structurally similar MBF1c proteins from *Nicotiana attenuata*, *Helianthus annuus*, *Arabidopsis thaliana*, *Triticum aestivum*, *Hordeum vulgare*, and *Hordeum brevisubulatum* were clustered in group 10. On the basis of the clustering patterns, we deduced that the MBF1 proteins evolved in a species-specific and conserved manner, and plant species of the same family or genus were strictly clustered together. The evolution of MBF1 proteins was relatively simple in monocots, but was more complex and diverse in dicots. Similar results have been reported for other examined proteins (Magwanga et al. 2018).

### Molecular characterization and the analysis of the *HbMBF1a* expression pattern

**Fig. 4:**
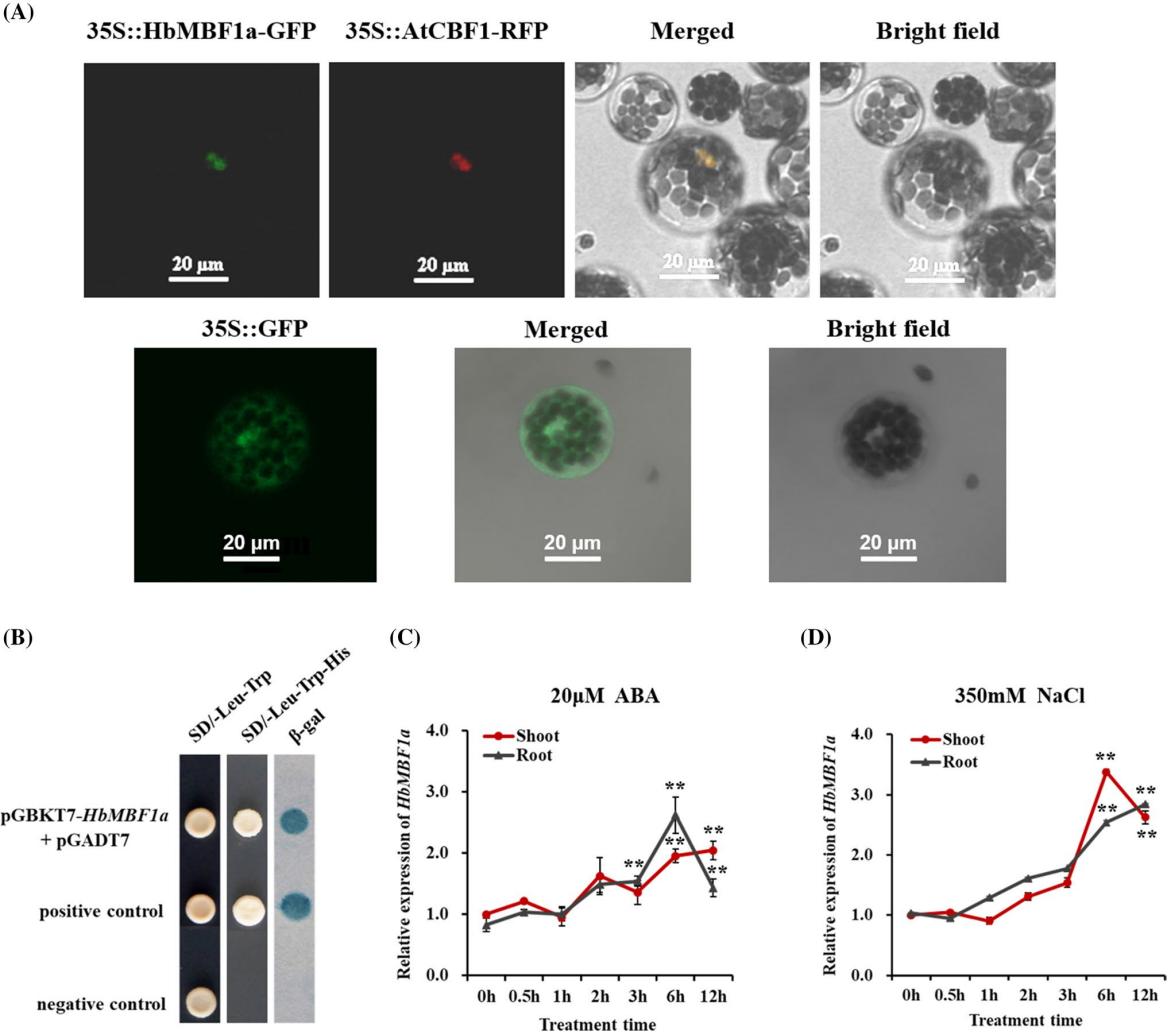
Analyses of the *HbMBF1a* expression patterns and the characteristics of the encoded protein. **a** Nuclear localization of the HbMBF1a-GFP fusion protein in *A. thaliana* mesophyll protoplasts. Bar = 20 μm. The *AtCBF1* gene encodes a nuclear localization marker, and the AtCBF1-RFP fusion protein was used as a nuclear localization control, 35S::GFP indicates the localization of empty vector GFP protein as a positive control. **b** Analysis of the transcriptional activation by HbMBF1a. Positive control: pGBKT7-53 and pGADT7-T co-transformation; negative control: pGBKT7-Lam and pGADT7-T co-transformation. **c** Expression pattern of *HbMBF1a* induced by 20 μM ABA. **d** Relative *HbMBF1a* expression trend in response to 350 mM NaCl. Three independent experiments were performed, and error bars indicate the standard deviation. Asterisks indicate significant differences according to Student’s *t*-test (*P < 0.05, **P < 0.01)

To examine the subcellular localization of the HbMBF1a protein, an *HbMBF1a*-*GFP* fusion construct was introduced into *A. thaliana* mesophyll cell protoplasts. The GFP signal was detected in the nucleus. Additionally, the AtCBF1-RFP fusion protein was used as a nuclear localization marker (Fig. 4a). To determine whether HbMBF1a functions as a transcription factor, we performed a transactivation assay in yeast, which confirmed that HbMBF1a can activate transcription (Fig. 4b). In this study, we used yeast cells co-transformed with the empty pGADT7 plasmid and the pGBKT7-*HbMBF1a* recombinant plasmid to verify transcriptional activation.

The expression levels of several *MBF1* family genes in various species are reportedly differentially induced by various abiotic stresses (Rizhsky et al. 2002; Tsuda and Yamazaki 2004; Kim et al. 2007). In this study, *HbMBF1a* expression was evaluated at various time-points after salt and ABA treatments. Transcript abundance was relatively high at 6 and 12 h after the salt and ABA treatments (Fig. 4c, d). The *HbMBF1a* expression pattern in response to salt stress was consistent with the transcriptome data (Fig. 2e). These results suggested that *HbMBF1a* expression is regulated by salt and exogenous ABA, but not during the early stage of the stress treatments.

### Overexpression of *HbMBF1a* enhances the salt tolerance of *Arabidopsis thaliana*

**Fig. 5:**
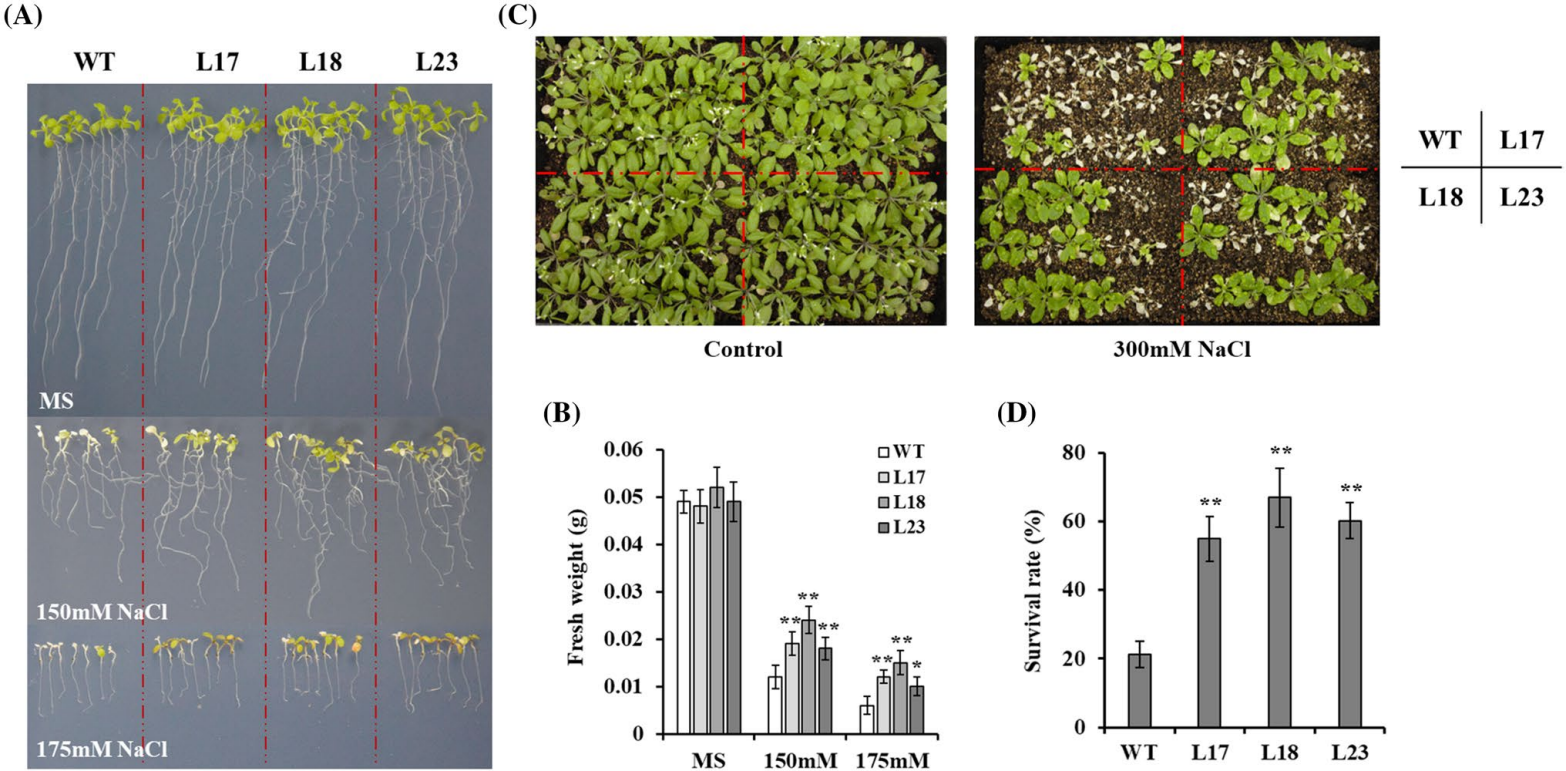
Phenotypic differences between *HbMBF1a*-overexpressing lines and wild-type plants exposed to salt stress. **a** Growth and development of the *HbMBF1a*-overexpressing lines and the wild-type plants in response to various salt treatments. Seven seedlings per line were examined. **b** Comparison of the fresh weights of the *HbMBF1a*-overexpressing lines and the wild-type plants following various salt treatments. The average fresh weight was calculated based on seven seedlings. Three independent experiments were performed, and error bars indicate the standard deviation. **c** Survival rates of the *HbMBF1a*-overexpressing lines and the wild-type plants under salt stress conditions. Twenty-one seedlings per line were analyzed. Control refers to the normal culture conditions without the salt treatment. The 23-day 300 mM NaCl treatment was followed by a 7-day recovery period (i.e., normal watering and culture conditions). **d** Comparison of the survival rates of the *HbMBF1a*-overexpressing lines and the wild-type plants under salt stress conditions. The *HbMBF1a*-overexpressing lines are represented by L17, L18, and L23. Three independent experiments were performed, and error bars indicate the standard deviation. Asterisks indicate significant differences according to Student’s *t*-test (*P < 0.05, **P < 0.01)

To further investigate the functions of HbMBF1a in plants, we generated *HbMBF1a*-overexpressing transgenic *A. thaliana* lines. We examined the growth and development of the *HbMBF1a*-overexpressing lines (L17, L18, and L23) and the WT control exposed to salt stress. The transgenic lines were more salt-tolerant than the WT plants following the 150 mM and 175 mM NaCl treatments. Additionally, compared with the WT plants, the *HbMBF1a*-overexpressing lines grew and developed better (Fig. 5a). The fresh weight of the *HbMBF1a*-overexpressing lines treated with 150 mM or 175 mM NaCl was significantly greater than that of the WT plants, especially following the 150 mM NaCl treatment (Fig. 5b). Moreover, the transgenic lines had higher germination and cotyledon greening rates than the WT control in response to the 125 mM and 150 mM NaCl treatments (Supplementary Fig. S3).

To confirm that the overexpression of *HbMBF1a* enhances the salt tolerance of *A. thaliana*, we analyzed the phenotype of the transgenic lines at the adult stage in addition to the seedling stage. Specifically, the *HbMBF1a*-overexpressing lines and the WT plants were grown in the greenhouse for 20 days, after which they were treated with 300 mM NaCl. The *HbMBF1a*-overexpressing lines were more salt tolerant than the WT plants during the adult stage (Fig. 5c). Normal watering and culture conditions were restored for 7 days after the 300 mM NaCl treatment. The survival rate of the *HbMBF1a*-overexpressing lines was significantly higher than that of the WT plants, indicating that the salt tolerance was enhanced (Fig. 5d).

### Transgenic *Arabidopsis thaliana* lines exhibited decreased sensitivity to exogenous ABA

**Fig. 6:**
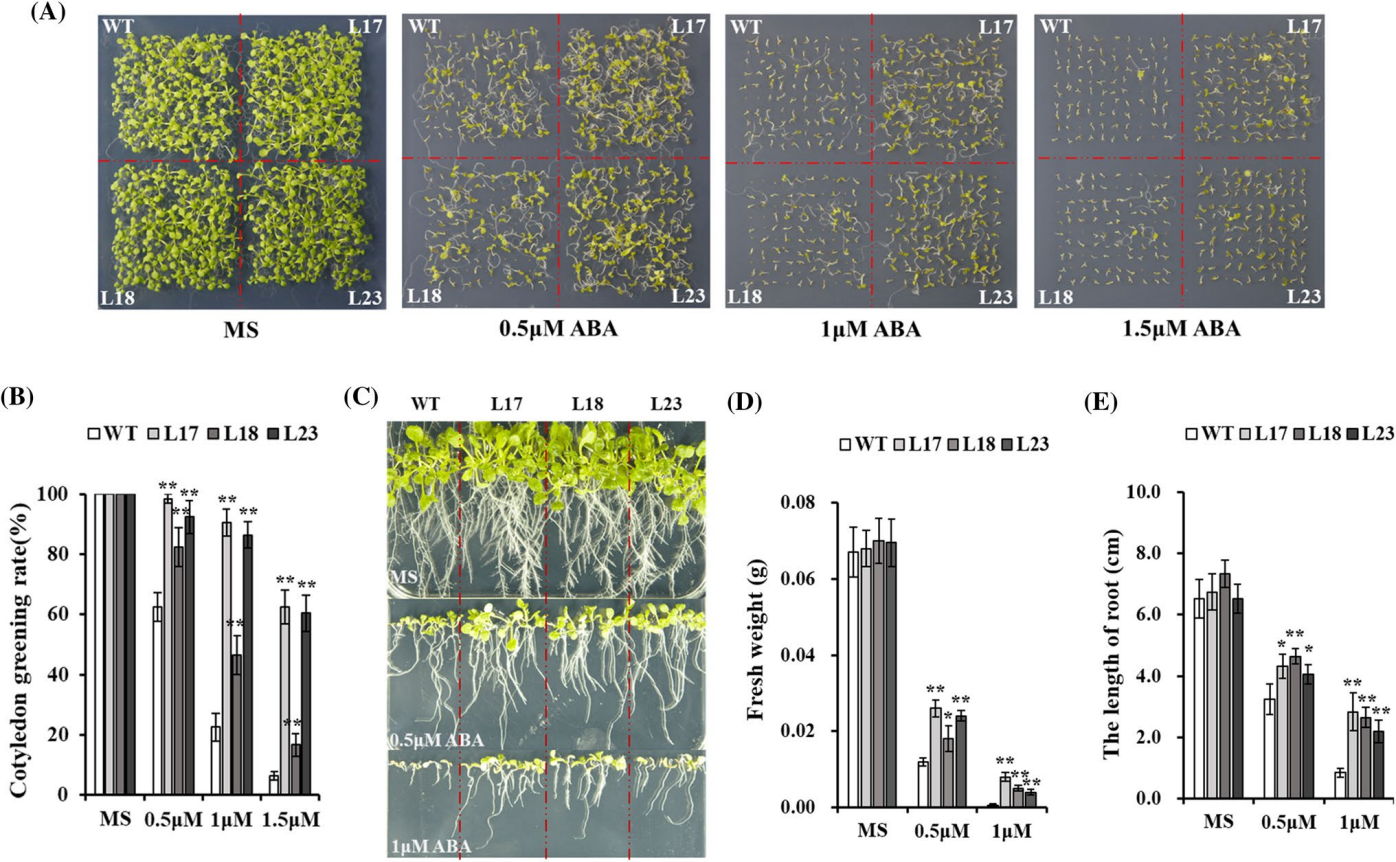
Sensitivity of the *HbMBF1a*-overexpressing lines and the wild-type plants to exogenous ABA. **a** Germination rate of the seeds (100 per line) of *HbMBF1a*-overexpressing lines and the wild-type plants in response to various ABA concentrations. **b** Comparison of the green cotyledon rate between the *HbMBF1a*-overexpressing lines and the wild-type plants following treatments with various ABA concentrations. Three independent experiments were performed, and error bars indicate the standard deviation. **c** Growth and development of the *HbMBF1a*-overexpressing lines and the wild-type plants exposed to ABA treatment. Seven seedlings per line were analyzed. **d** Comparison of the fresh weight between *HbMBF1a*-overexpressing lines and the wild-type plants in response to various ABA concentrations. The average fresh weight was calculated based on seven plants, and three independent experiments were performed. **e** Root lengths of the *HbMBF1a*-overexpressing lines and the WT plants exposed to various ABA concentrations. Three independent experiments were performed, and error bars indicate the standard deviation. Asterisks indicate significant differences according to Student’s *t*-test (*P < 0.05, **P < 0.01)

The ABA signal transduction pathway is involved in a variety of abiotic stress responses in plants. Therefore, we also tested the sensitivity of *HbMBF1a*-overexpressing *A. thaliana* plants to exogenous ABA. We sowed *A. thaliana* seeds (100 per line) individually on MS medium containing various ABA concentrations. The germination and cotyledon greening rates were compared between the *HbMBF1a*-overexpressing lines and the WT plants (Fig. 6a). The transgenic *A. thaliana* plants were less sensitive to exogenous ABA than the WT plants. Additionally, the germination rate of the *HbMBF1a*-overexpressing lines was significantly higher than that of the WT plants, as was the cotyledon greening rate (Fig. 6b).

We examined the growth and development of *HbMBF1a*-overexpressing lines and the WT plants following an ABA treatment. The results suggested that *HbMBF1a* overexpression may enhance the adaptability of *A. thaliana* to exogenous ABA. Compared with the WT plants, the *HbMBF1a*-overexpressing lines maintained relatively stable growth and development (Fig. 6c). The fresh weight of *HbMBF1a*-overexpressing lines was significantly greater than that of the WT plants, especially in response to 1 μM ABA (Fig. 6d). Moreover, the roots of the transgenic lines were significantly longer than the WT roots, particularly following the 1 μM ABA treatment (Fig. 6e). Thus, the ABA signal transduction pathway likely influences the HbMBF1a-mediated salt stress response in *A. thaliana*.

### Overexpression of *HbMBF1a* affects the expression of salt- and ABA-responsive genes

**Fig. 7:**
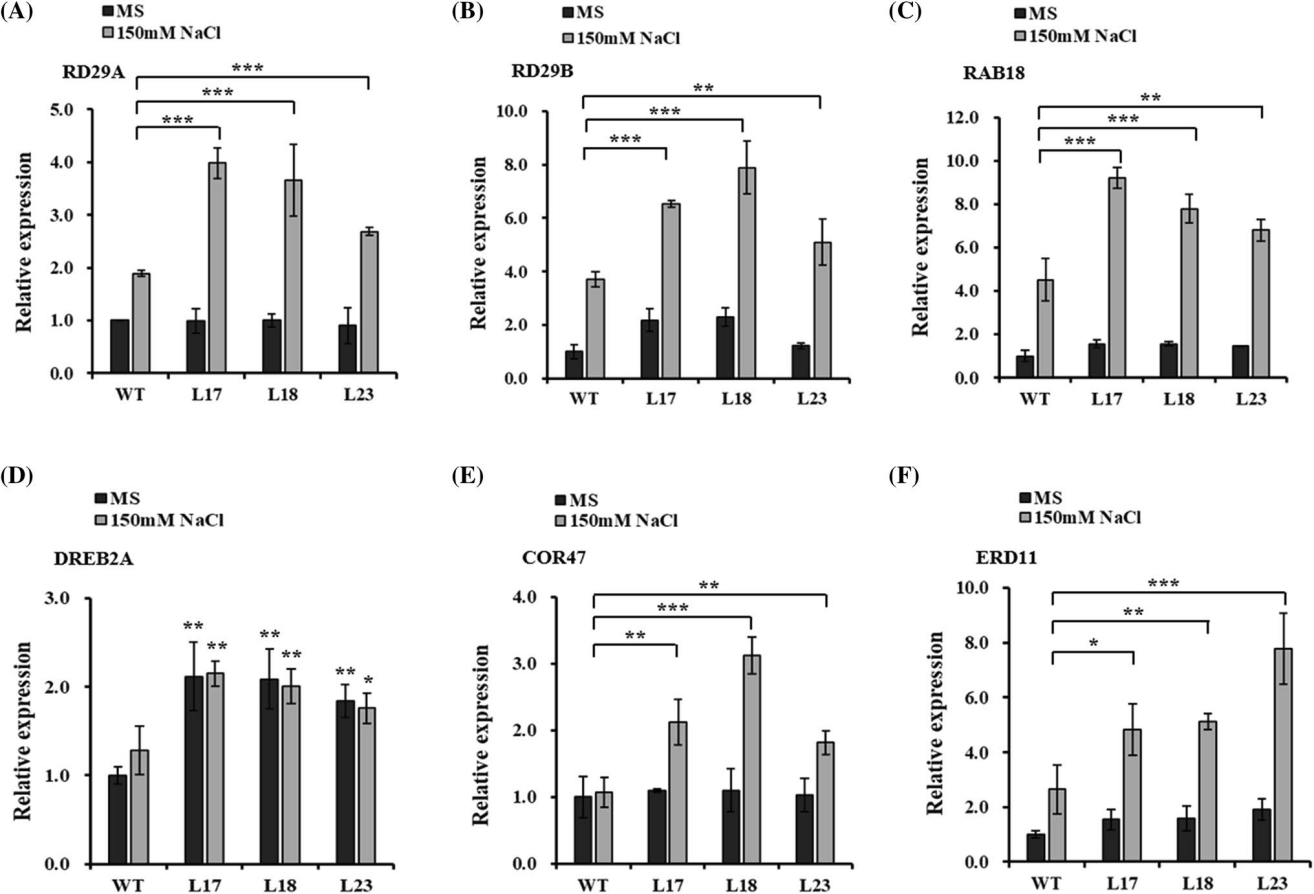
Expression analysis of abiotic stress-related genes in the *HbMBF1a*-overexpressing lines and the WT plants following the 150 mM NaCl treatment. **a**–**f** Relative *AtRD29A*, *AtRD29B*, *AtRAB18*, *AtDREB2A*, *AtCOR47*, and *AtERD11* transcript levels. After a 7-day growth on MS medium, the seedlings were transferred to MS medium containing 150 mM NaCl and incubated for 2 days. Three independent experiments were performed, and error bars indicate the standard deviation. Asterisks indicate significant differences according to Student’s *t*-test (*P < 0.05, **P < 0.01, ***P < 0.001)

We determined that the overexpression of *HbMBF1a* in *A. thaliana* positively regulates the salt-response pathway and ABA signaling. We predicted that HbMBF1a may positively regulate the expression of salt- and ABA-responsive genes. To confirm this, we analyzed the expression of 13 key genes in salt- and ABA-induced responses. We analyzed gene expression levels by qRT-PCR. There were no significant differences in the relative gene expression levels between the *HbMBF1a*-overexpressing lines and the WT plants grown on MS medium under normal conditions. However, after a 2-day NaCl treatment, the relative gene expression levels were higher in the transgenic lines than in the WT plants. Notably, *DREB2A* expression levels varied between the *HbMBF1a*-overexpressing lines and the WT plants, regardless of whether the MS medium was supplemented with NaCl (Fig. 7).

**Fig. 8:**
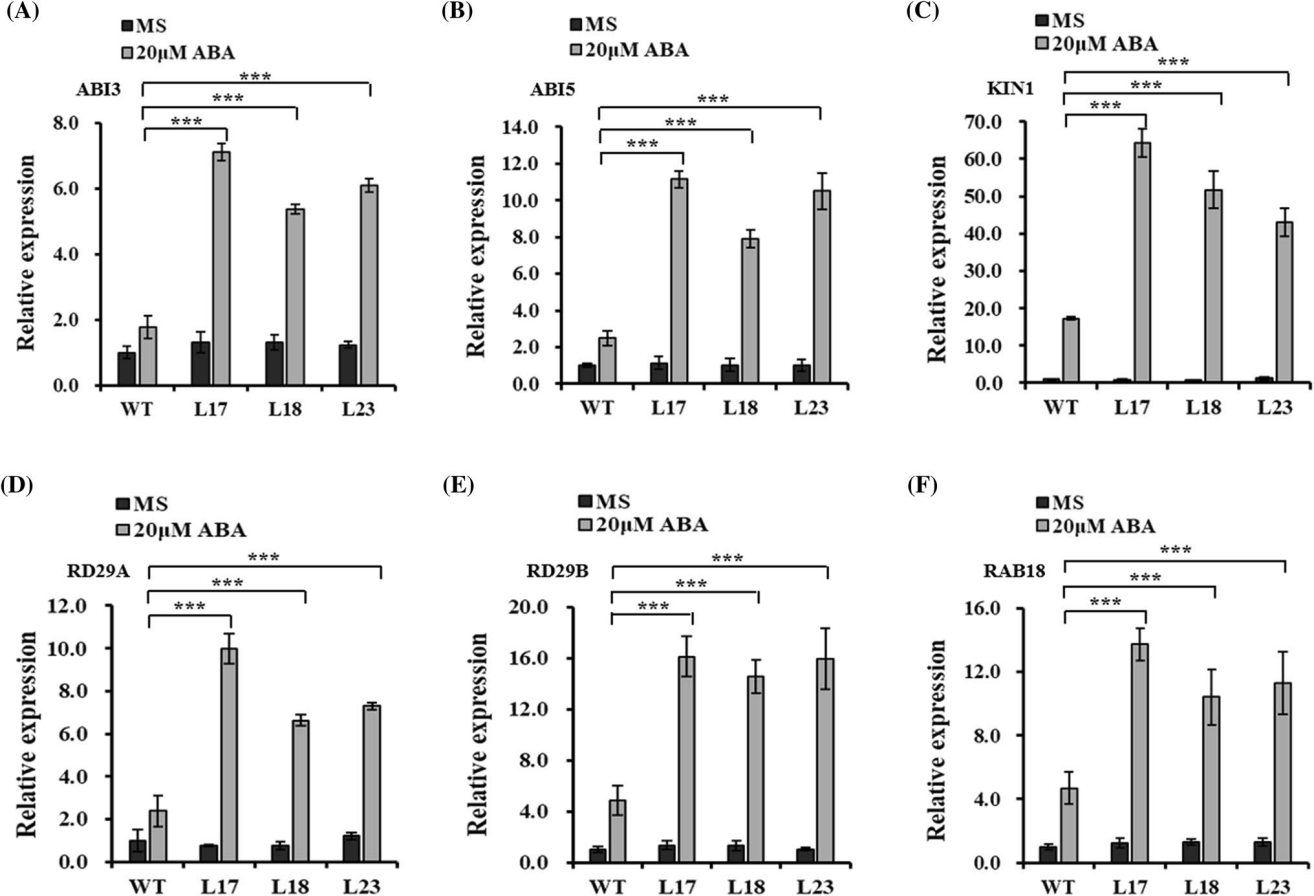
Expression analysis of abiotic stress-related genes in *HbMBF1a*-overexpressing lines and the WT plants treated with 20 μM ABA. **a**–**f** Relative *AtABI3*, *AtABI5*, *AtKIN1*, *AtRD29A*, *AtRD29B*, and *AtRAB18* transcript levels. After a 7-day growth on MS medium, the seedlings were transferred to MS medium containing 20 μM ABA and incubated for 2 days. Three independent experiments were performed, and error bars indicate the standard deviation. Asterisks indicate significant differences according to Student’s *t*-test (***P < 0.001)

We also confirmed that the *AtABI3*, *AtABI5*, *AtKIN1*, *AtRD29A*, *AtRD29B*, and *AtRAB18* expression levels were significantly up-regulated by the 20 μM ABA treatment. Moreover, on MS medium with 20 μM ABA, the relative expression levels of these genes were significantly higher in the *HbMBF1a*-overexpressing lines than in the WT plants. In contrast, the expression levels of these genes were not significantly different between the transgenic and WT plants on MS medium alone (i.e., no ABA) (Fig. 8). These results prove that HbMBF1a positively regulates ABA signaling.

We examined the expression of 13 key genes associated with abiotic stress responses. Some of these genes were not differentially expressed between the *HbMBF1a*-overexpressing lines and the WT plants following the salt or ABA treatments. Examples include the *AtRD22*, *AtABI3*, *AtABI5*, *AtNCED3*, *AtKIN1*, and *AtWIN1* genes following the 150 mM NaCl treatment. Additionally, the expression levels of *AtRD22*, *AtCOR47*, *AtERD11*, *AtDREB2A*, *AtNCED3*, and *AtWIN1* were not significantly different between the *HbMBF1a*-overexpressing lines and the WT plants in response to 20 μM ABA. Since the expression of ABA response-related genes was basically unchanged under salt stress, and the expression of several stress-response-related genes did not change under ABA treatment. In summary, it suggested that *HbMBF1a* was involved in the regulation of salt stress response and ABA sensitivity in *Arabidopsis thaliana*. Besides, because the three primer pairs specific for *ABI4* were unable to efficiently and specifically generate amplification products, we were unable to analyze the *ABI4* expression level under salt and ABA treatments.

Specially, the expression of *AtDREB2A*, *AtCOR47*, *AtERD11*, *AtRD29A*, *AtRD29B* and *AtRAB18*, which are also drought-inducible genes, were significantly higher in *HbMBF1a*-overexpressing lines than in wild-type plants. Therefore, we also observed the drought stress tolerance of *HbMBF1a*-overexpressing lines, and found that *HbMBF1a* overexpression can enhance the drought tolerance of *Arabidopsis*. The survival rate of *HbMBF1a*-overexpressing lines was also significantly higher than that of wild type after rehydration (Supplementary Fig. S4).

## Discussions

Plant transcriptome sequencing has been widely used to discover genes, develop markers, and analyze of the gene regulatory networks, particularly for those of non-model organisms without a reference genome (Udomchalothorn et al. 2017; Lu et al. 2018). There are several published reports describing the transcriptome regulation of barley and wild barley (*H. spontaneum*) grown under salt stress conditions (Bahieldin et al. 2015; Hill et al. 2016). However, the transcriptome changes and key regulatory genes expressed in response to saline conditions remain largely unknown for wild *Hordeum* species.

An analysis involving the STEM program is an increasingly popular method for studying diverse biological processes. Specifically, the STEM program is designed for clustering, comparing, and visualizing gene expression data from short time-series microarray experiments (approximately eight of fewer time-points) (Ernst and Bar-Joseph 2006). Multiprotein bridging factor 1 mediates the transcriptional activity of transcription factors via direct interactions (Takemaru et al. 1997). The MBF1 protein contains an N-terminal domain comprising about 50 amino acids, a conserved HTH domain, and a short C-terminus (Fig. 3a). The N- and C-terminal sequences vary among different organisms, whereas the HTH domain, which contains four α-helices, is conserved (de Koning et al. 2009). The HTH domain is responsible for the MBF1 function, and differences at the N- and C-termini do not affect the main MBF1 activities (Ozaki et al. 1999).

The function of MBF1 during salt stress responses remains unclear. However, its function as a transcriptional co-activator has been confirmed based on thorough studies of several eukaryotic proteins, which have often indicated MBF1 contributes to responses to heat and oxidative stresses (Qin et al. 2015; Chu et al. 2018). Interestingly, algae have only one *MBF1* gene, in contrast to most land plants, which contain at least two types of *MBF1* genes, suggesting a gene duplication event occurred early in the evolution of land plants (Alavilli et al. 2017). Within the MBF1c clade, MBF1c orthologs of all vascular plant species (i.e., monocots and dicots) were clustered together, implying MBF1c proteins vary considerably from the other MBF1 family proteins (Fig. 3b). Accordingly, plant MBF1c proteins may possess non-redundant functions that differ from those of MBF1a/b proteins, despite sharing similar conserved domains with other MBF1 family proteins. Similar results were obtained in a previous study of *MBF1c* (Tsuda and Yamazaki 2004; Alavilli et al. 2017).

Genomic characteristics reflect the adaptability of plants to adverse conditions. Moreover, identifying the genes involved in the stress tolerance of plants will help to characterize the underlying mechanism. In this study, we determined that HbMBF1a from the halophyte *H. brevisubulatum* is a nuclear protein and can activate transcription (Fig. 4a, b). The same conclusions were reached in another study (Wang et al. 2017). These findings imply that HbMBF1a can act as a transcriptional coactivator that regulates the transcription of downstream gene targets. The study has found that MBF1 genes activate transcriptional accumulation of transcription factors WRKY and CBF-like as well as MAPK3/11 and calcium-binding proteins (Suzuki et al. 2005). bZIP-type transcription factor is specifically associated with BbMBF1-interactome under oxidation (Song et al. 2015). *AtMBF1* *s* regulate the expression of the ABR1 gene during normal growth and stress conditions (Arce et al. 2010). In addition, we also confirmed that HbMBF1a and HbWRKY family gene interact to regulate the transcriptional expression of *HbWRKY* gene.

We observed that the *HbMBF1a* expression level was up-regulated more than twofold at 6 and 12 h compared with the expression level at 0 h following the 350 mM NaCl treatment (Fig. 4d). Regarding the effects of the 20 μM ABA treatment, the *HbMBF1a* expression level was significantly higher at 3, 6, and 12 h than at 0 h (Fig. 4c). Previous studies proved that *MBF1* expression is significantly enhanced by abiotic stresses in plants (Wang et al. 2017; Arce et al. 2010) as well as in bacteria and fungi (Fan et al. 2017; Coto et al. 2011). However, the expression trends of *MBF1*-like genes are not static. Rather, they vary depending on the stress and the organism. Earlier investigations confirmed that *CaMBF1* expression can be suppressed by SA, salt, osmotic, and heavy metal stresses, and the overexpression of *CaMBF1* in *A. thaliana* negatively affects the tolerance to cold and high salt stresses (Guo et al. 2014).

The *MBF1* genes are involved in the tolerance of plants to biotic and abiotic stresses. In previous studies, the overexpression of the Antarctic moss *MBF1c* gene in *A. thaliana* enhanced salt tolerance (Alavilli et al. 2017), and the constitutive expression of the maize *MBF1a* gene in *A. thaliana* also increased salt tolerance (Kim et al. 2007). These results are consistent with the enhanced salt tolerance of *HbMBF1a*-overexpressing *A. thaliana* plants under saline conditions in the current study (Fig. 5a, c). Although *MBF1* genes have been detected in many organisms, most of them have yet to be functionally characterized. However, a previous study revealed that *AtMBF1c* expression is highly induced by heat and drought stresses (Rizhsky et al. 2004). This gene is the most studied of the three *AtMBF1* genes (*AtMBF1a*, *b*, and *c*), and encodes a key regulator of heat stress response networks (Suzuki et al. 2008).

Initially, *MBF1* genes were associated with the ethylene signal transduction pathway (Zegzouti et al. 1999). Unlike the roots of ethylene insensitive mutants (*ein2*, *ein3*, and *etr1*) (Ghassemian et al. 2000), the growth of the *abc* mutant (triple knock-down of *MBF1* genes) roots was unaffected by exogenous ABA, suggesting that MBF1 may modulate specific ABA-dependent responses (Mauro et al. 2012). However, our study revealed that the roots of *HbMBF1a*-overexpressing lines were significantly longer than those of the WT plants under exogenous ABA conditions (Fig. 6c, e). Furthermore, three stress-related genes, RD29A, RD29B, RAB18, were collectively up-regulated by both NaCl and ABA treatments. But, further studies are needed to determine whether *HbMBF1a* participates in salt tolerance regulation through ABA-dependent or ABA-independent pathways. *A. thaliana* overexpressing *ERF4* are reportedly less sensitive to ABA than WT plants, but are hypersensitive to osmotic stress (Yang et al. 2005). Because ABA may control the biosynthesis, catabolism, or signaling of ethylene and vice versa to enhance abiotic stress tolerance, the influence of MBF1 on this apparent hormonal cross-talk should be explored.

Analyzing the expression levels of specific marker genes under abiotic stress conditions is important for assessing the stress resistance of transgenic lines. Previous studies have included many examples of the functional analysis of the overexpression of genes in *A. thaliana*. Some of these studies have confirmed that the expression levels of marker genes related to stress responses are significantly up- or down-regulated in transgenic lines exposed to abiotic stresses (Zhao et al. 2018; Feng et al. 2019). Another study involved a GO enrichment analysis of salt-regulated genes (Alavilli et al. 2017). Some studies have analyzed the expression of stress-related genes in transgenic yeast (Wang et al. 2017; Chu et al. 2018). The regulation of target gene expression can also be determined by analyzing the expression of stress response genes in *A. thaliana* mutants (Zandalinas et al. 2016). In summary, future investigations will need to analyze the relative expression-level differences in the salt and ABA response-related marker genes between the *HbMBF1a* transgenic lines and the WT plants. Furthermore, the regulatory roles of *HbMBF1a* should be elucidated.

## Conclusions

In this study, we isolated and characterized *HbMBF1a* from a transcriptome database for *H. brevisubulatum* exposed to salt stress. We subsequently examined the following in *H. brevisubulatum*: the classification and expression profiles of the differentially expressed genes, *HbMBF1* family members; phylogenetic relationships among MBF1 proteins, domain features of MBF1 proteins and the tissue-specific *HbMBF1a* expression patterns in response to salt and ABA treatments. Since the discovery of *MBF1* genes, there has been relatively little published research on the *MBF1* genes in Gramineae species. To verify that HbMBF1a helps regulate stress responses, we overexpressed *HbMBF1a* in *A. thaliana* and observed that the resulting transgenic lines were highly salt tolerant and ABA insensitive. Moreover, the expression levels of stress-related genes were significantly up-regulated in *HbMBF1a*-overexpressing lines. This finding may be useful for clarifying the molecular mechanism underlying the enhanced stress resistance. The abundance of *HbMBF1* genes and the unique gene structures reported herein provide a solid foundation for future studies aimed at elucidating the evolution of the *MBF1* gene family and the potential functions of HbMBF1a in plants under high salt and ABA conditions.

## Electronic supplementary material

Below is the link to the electronic supplementary material.

Supplementary material 1 (TIFF 1160 kb)

Supplementary material 2 (TIFF 56 kb)

Supplementary material 3 (TIFF 3335 kb)

Supplementary material 4 (TIFF 909 kb)

Supplementary material 5 (DOCX 17 kb)

Supplementary material 6 (XLSX 16 kb)

Supplementary material 7 (XLSX 21 kb)

Supplementary material 8 (DOCX 14 kb)
