# Supplementary figures and images for: Overexpression of HbMBF1a, encoding multiprotein bridging factor 1 from the halophyte Hordeum brevisubulatum, confers salinity tolerance and ABA insensitivity to transgenic Arabidopsis thaliana
Source: Plant Mol Biol. 2019 Oct 26;102(1):1–17. doi: 10.1007/s11103-019-00926-7 (PMC6976555; doi:10.1007/s11103-019-00926-7)

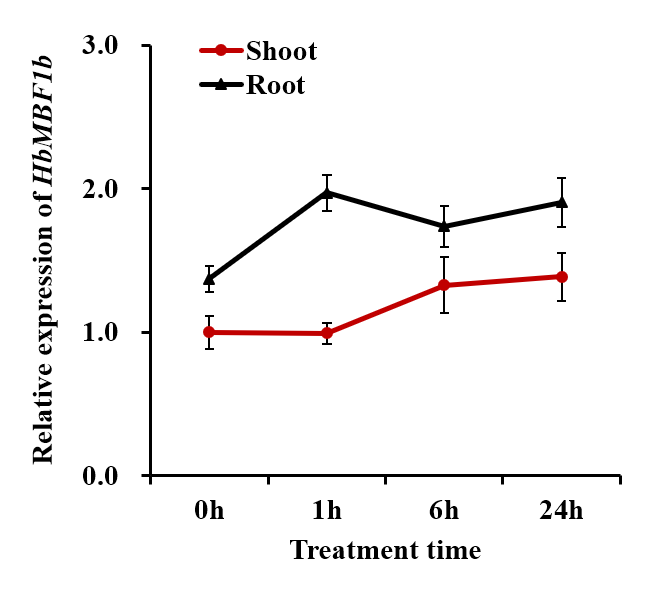

Supplement: Supplementary file 1 — Supplementary material 1 (TIFF 1160 kb) [file 11103_2019_926_MOESM1_ESM.tif]

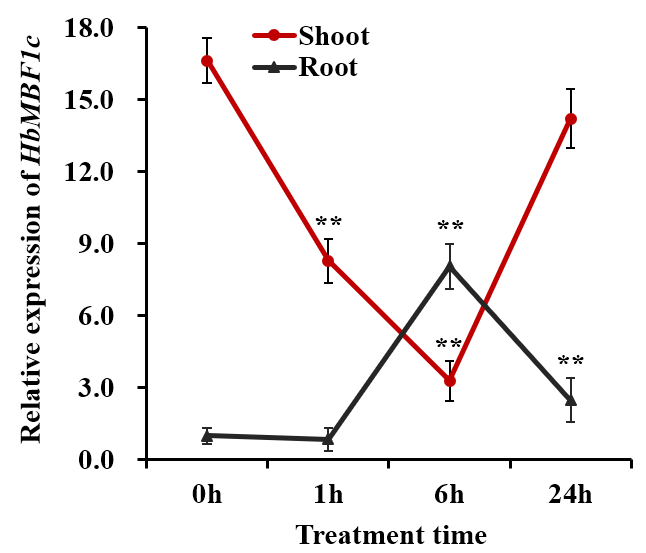

Supplement: Supplementary file 2 — Supplementary material 2 (TIFF 56 kb) [file 11103_2019_926_MOESM2_ESM.tif]

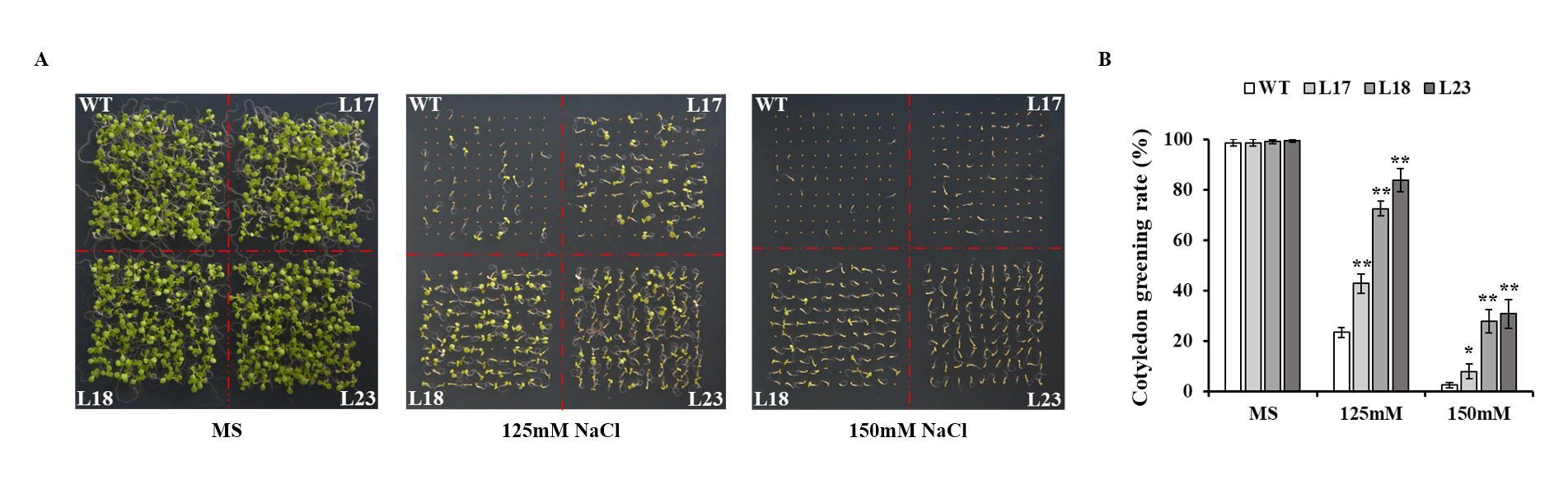

Supplement: Supplementary file 3 — Supplementary material 3 (TIFF 3335 kb) [file 11103_2019_926_MOESM3_ESM.tif]

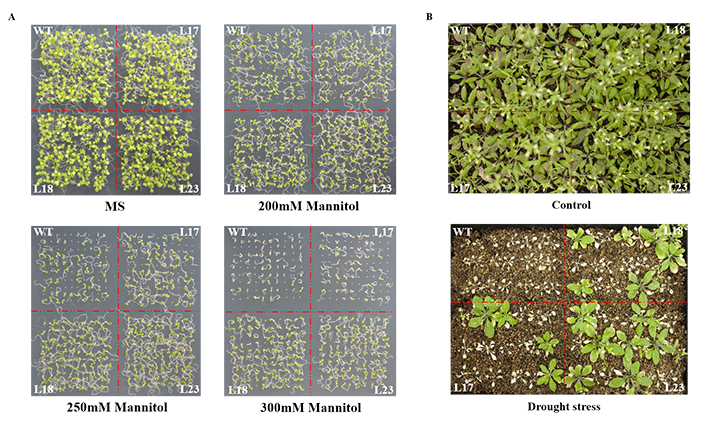

Supplement: Supplementary file 4 — Supplementary material 4 (TIFF 909 kb) [file 11103_2019_926_MOESM4_ESM.tif]
