# Supplementary material for: Overexpression of HbMBF1a, encoding multiprotein bridging factor 1 from the halophyte Hordeum brevisubulatum, confers salinity tolerance and ABA insensitivity to transgenic Arabidopsis thaliana
Source: Plant Mol Biol. 2019 Oct 26;102(1):1–17. doi: 10.1007/s11103-019-00926-7 (PMC6976555; doi:10.1007/s11103-019-00926-7)
Supplement: Supplementary file 5 — Supplementary material 5 (DOCX 17 kb) [file 11103_2019_926_MOESM5_ESM.docx]

Supplementary table 1: List of *HbMBF1a* and other genes specific primers in this study.

| Purpose | Gene | Forward primer (5' to3') | Reverse primer (3' to 5') |
| --- | --- | --- | --- |
| Correction | *HbMBF1a* | ATGGCTGGGATTGGTCCGCTCAG | TTACTTCTTGCTACGCAGCTTAGCTCC |
| Location | *HbMBF1a* | TGGATCCATGGCTGGGATTGGTCC | AGAGCTCCTTCTTGCTACGCAGCTTA |
| Transgene | *HbMBF1a* | TTCTAGAATGGCTGGGATTGGTCC | ACCCGGGCTTCTTGCTACGCAGC |
| Identification | *HbMBF1a* | ATCCAGGAGTACGAATCGGG | ATCAACCAACAGGAACAGCC |
| Transcriptional activation | *HbMBF1a* | TGGATCCATGGCTGGGATTGGTC | ACTGCAGTTACTTCTTGCTACGCAGC |
| Transcriptional activation | *AtCBF1* | TGGATCCATGAACTCATTTTCAGCTTTTTCTG | TGGTACCGTAACTCCAAAGCGACAC |
| qRT-PCR | *HbADP* | CAAGAGATAGTATGTGTGCGTATG | AACCACGGCACAGAAATACTGAT |
| qRT-PCR | *HbUBI* | TGGATGTTGTAGTCGGCGAG | ACGTCAAGGCCAAGATCCAG |
| qRT-PCR | *HbMBF1a* | GATAGTGGTGCGGAAGAAGGCGCAG | TGTTCCAGCGTTGTACTTCTTGGTG |
| qRT-PCR | *AtSAND* | AACTCTATGCAGCATTTGATCCACT | TGATTGCATATCTTTATCGCCATC |
| qRT-PCR | *AtABI3* | CACAGCCAGAGTTCCTTCCTTTACT | TAGTTGCTGAGGAACACAAACGG |
| qRT-PCR | *AtABI4* | GGGCAGGAACAAGGAGGAAGTG | TCTCCTCCAAAAGGCCAAATGGT |
| qRT-PCR | *AtABI4* | TGGGTCTGGATCCGGGTTAT | CCAATGCTCTTGACCGACCT |
| qRT-PCR | *AtABI4* | CGATTCCACCACCGACTCAT | CCACGGTAACGGAACTTGGA |
| qRT-PCR | *AtABI5* | ATGATCAAGAACCGCGAGTCTGC | CGGTTGTGCCCTTGACTTCAAAC |
| qRT-PCR | *AtRAB18* | GGCTTGGGAGGAATGCTTCA | CGCTTGAGCTTGACCAGACT |
| qRT-PCR | *AtRD29A* | GGAAGTGAAAGGAGGAGGAGGAA | CACCACCAAACCAGCCAGATG |
| qRT-PCR | *AtRD29B* | GAATCAAAAGCTGGGATGGA | TGCTCTGTGTAGGTGCTTGG |
| qRT-PCR | *AtRD22* | GTCAGGGCTGTTTCCACTGA | AACACCGCGAATGGGTACTT |
| qRT-PCR | *AtCOR47* | GAAGATCAAGGAGAAGCTTCCT | TTCTTCACTTCCTCTTCAGTGG |
| qRT-PCR | *AtKIN1* | CAACAGGCGGGAAAGAGTGTA | TTTGACCCGAATCGCTACTTG |
| qRT-PCR | *AtDREB2A* | AAGGAGGACCAGAGAATAG | GCCAAAGGACCATACATAG |
| qRT-PCR | *AtERD11* | CCCCTTTGGTAAAGTTCC | ATGTCCTTGCCAGTTGAG |
| qRT-PCR | *AtNCED3* | GCTGCGGTTTCTGGGAGAT | TTGAGAAGACGATAATGGCG |
| qRT-PCR | *AtWIN1* | CTTCATCGCTCTCTTCCATCC | CCAATACTTCTTCTCTGCTGC |

The sequence marked in red indicates the cleavage site.
