# Supplementary material for: Overexpression of HbMBF1a, encoding multiprotein bridging factor 1 from the halophyte Hordeum brevisubulatum, confers salinity tolerance and ABA insensitivity to transgenic Arabidopsis thaliana
Source: Plant Mol Biol. 2019 Oct 26;102(1):1–17. doi: 10.1007/s11103-019-00926-7 (PMC6976555; doi:10.1007/s11103-019-00926-7)
Supplement: Supplementary file 8 — Supplementary material 8 (DOCX 14 kb) [file 11103_2019_926_MOESM8_ESM.docx]

The corrected nucleotide sequence of *HbMBF1a* (The yellow label shows the CDS):

TTTGCTGGCCTTTTGCTCACATGTTCTTTCCTGCGTTATCCCCTGATTTCTGTGGATAACCGTATTACCGCCTTTGAGTGAGCTGATACCGCTCGCCGCAGCCGAACGACCGAGCGCAGCGAGTCAGTGAGCGAGGAAGCGGAAGAGCGCCCAATACGCAAACCGCCTCTCCCCGCGCGTTGGCCGATTCATTAATGCAGCTGGCACGACAGGTTTCCCGACTGGAAAGCGGGCAGTGAGCGCAACGCAATTAATGTGAGTTAGCTCACTCATTAGGCACCCCAGGCTTTACACTTTATGCTTCCGGCTCGTATGTTGTGTGGAATTGTGAGCGGATAACAATTTCACACAGGAAACAGCTATGACCATGATTACGCCAAGCTCAGAATTAACCCTCACTAAAGGGACTAGTCCTGCAGGTTTAAACGAATTGCCCTTTATGGCTGGGATTGGTCCGATCAGGCAGGACTGGGAGCCGATAGTGGTGCGGAAGAAGGCGCAGAACGCCGCGGACAAGAAGGACGAAAAGGCCGTCAACGCTGCCCGCCGCTCCGGCGCCGAGATCGACACCACCAAGAAGTACAACGCTGGAACAAACAAGGCTGCATCTAGCGGAACTTCCCTCAACACCAAGCGGCTCGACGACGACACCGAGAACCTTTCCCATGAGCGTGTTTCAAGTGACCTGAAGAAAAACCTGATGCAAGCAAGGCTGGATAAGAAGATGACACAGGCACAACTTGCTCAGATGATCAATGAGAAGCCACAGGTGATCCAGGAGTACGAATCGGGCAAGGCAATTCCGAACAATCAGATAATTGGAAAGCTCGAGAGGGCACTTGGAGCTAAGCTGCGTAGCAAGAAGTAATAAGGGCAATTCGCGGCCGCTAAATACTAATGGGCCACTGTACTTCCACCGAATG

The amino acid sequence of HbMBF1a:

MAGIGPIRQDWEPIVVRKKAQNAADKKDEKAVNAARRSGAEIDTTKKYNAGTNKAASSGTSLNTKRLDDDTENLSHERVSSDLKKNLMQARLDKKMTQAQLAQMINEKPQVIQEYESGKAIPNNQIIGKLERALGAKLRSKK
